# Impact of COVID-19 pandemic in the Brazilian maternal mortality ratio: A comparative analysis of Neural Networks Autoregression, Holt-Winters exponential smoothing, and Autoregressive Integrated Moving Average models

**DOI:** 10.1371/journal.pone.0296064

**Published:** 2024-01-31

**Authors:** Mayara Carolina Cañedo, Thiago Inácio Barros Lopes, Luana Rossato, Isadora Batista Nunes, Izadora Dillis Faccin, Túlio Máximo Salomé, Simone Simionatto

**Affiliations:** Laboratório de Pesquisa em Ciências da Saúde, Universidade Federal da Grande Dourados, Dourados, MS, Brazil; King Abdulaziz City for Science and Technology (KACST), SAUDI ARABIA

## Abstract

**Background and objectives:**

The acute respiratory infection caused by severe acute respiratory syndrome coronavirus disease (COVID-19) has resulted in increased mortality among pregnant, puerperal, and neonates. Brazil has the highest number of maternal deaths and a distressing fatality rate of 7.2%, more than double the country’s current mortality rate of 2.8%. This study investigates the impact of the COVID-19 pandemic on the Brazilian Maternal Mortality Ratio (BMMR) and forecasts the BMMR up to 2025.

**Methods:**

To assess the impact of the COVID-19 pandemic on the BMMR, we employed Holt-Winters, Autoregressive Integrated Moving Average (ARIMA), and Neural Networks Autoregression (NNA). We utilized a retrospective time series spanning twenty-five years (1996–2021) to forecast the BMMR under both a COVID-19 pandemic scenario and a controlled COVID-19 scenario.

**Results:**

Brazil consistently exhibited high maternal mortality values (mean BMMR [1996–2019] = 57.99 ±6.34/100,000 live births) according to World Health Organization criteria. The country experienced its highest mortality peak in the historical BMMR series in the second quarter of 2021 (197.75/100,000 live births), representing a more than 200% increase compared to the previous period. Holt-Winter and ARIMA models demonstrated better agreement with prediction results beyond the sample data, although NNA provided a better fit to previous data.

**Conclusions:**

Our study revealed an increase in BMMR and its temporal correlation with COVID-19 incidence. Additionally, it showed that Holt-Winter and ARIMA models can be employed for BMMR forecasting with lower errors. This information can assist governments and public health agencies in making timely and informed decisions.

## Introduction

The acute respiratory infection caused by severe acute respiratory syndrome coronavirus 2 (SARS-CoV-2) has led to increased mortality among pregnant, puerperal, and neonates [1]. Maternal deaths related to coronavirus disease (COVID-19) in Brazil have exceeded global figures due to factors such as clinical characteristics, social conditions, and barriers to adequate care [2]. According to the COVID-19 Observatory Newsletter, Brazil has the highest number of maternal deaths and a distressing fatality rate of 7.2%, more than double the country’s current mortality rate of 2.8% [3]. A study in Brazil reported 9,609 cases of pregnant and puerperal individuals with SARS-CoV-2 from December 2019 to August 2020, of which 4,230 (44.0%) were confirmed as COVID-19 positive. Among these cases, 533 (12.60%) of pregnant and puerperal individuals died, with 354 (64.0%) of these deaths attributed to COVID-19 [4]. A study conducted in the Democratic Republic of Congo revealed that most COVID-19-related deaths were due to delayed care and advanced age, with common causes, including hemorrhages, uterine ruptures, infections, and dystocias [5].

Access to quality prenatal, birthing, and postpartum care was compromised for pregnant and puerperal women during the SARS-CoV-2 pandemic [4]. In 2021, pregnant and puerperal women faced unfavorable outcomes and severe clinical presentations related to COVID-19 due to the presence of high-risk SRAG symptoms and a greater risk of intubation and intensive care unit (ICU) admission compared to other groups [6]. Additionally, pregnant women encountered challenges in accessing ventilators and intensive care during the pandemic [3].

Contingency measures focused on maternal health are essential to enhance access to prenatal services and intensive care for pregnant, puerperal women, and neonates [7]. The World Health Organization (WHO) emphasizes the need to reduce maternal mortality by 2030, as outlined in the Sustainable Development Goals (SDGs) [8]. However, barriers to accessing specialized care services and appropriate monitoring of obstetric complications persist in Brazilian hospitals and primary care settings [1].

Maternal mortality values over the years can be analyzed as a time series, and forecasting methods utilize time series data to predict future trends. Two widely recognized time series models for forecasting COVID-19 deaths and recoveries are the Holt-Winters and Auto-Regressive Integrated Moving Average (ARIMA) models [9]. The application of novel forecasting methods, including Neural Network Autoregression (NNA), has gained popularity. NNA mimics the structure and functionality of the human brain, using “neurons” to address complex challenges [10]. NNAs have been successfully employed in various fields to predict clinical events, such as peripherally inserted central catheter-related thrombosis in breast cancer patients [11].

Hence, the application of forecasting methods offers a quantitative framework for scientists to explore hypotheses related to potential disease mechanisms or causes of mortality. To the best of our knowledge, this is the first published comparative study, including Holt-Winters, ARIMA, and NNA models, on Brazilian Maternal Mortality Ratios (BMMR). These models are valuable for assessing the impact of interventions, optimizing the effectiveness of control strategies, and generating short- and long-term forecasts. Therefore, this study aims to analyze the relationship between BMMR and the COVID-19 pandemic and forecast BMMR trends up to 2025.

### Background Brazilian Maternal Mortality Ratios

**Fig 1:**
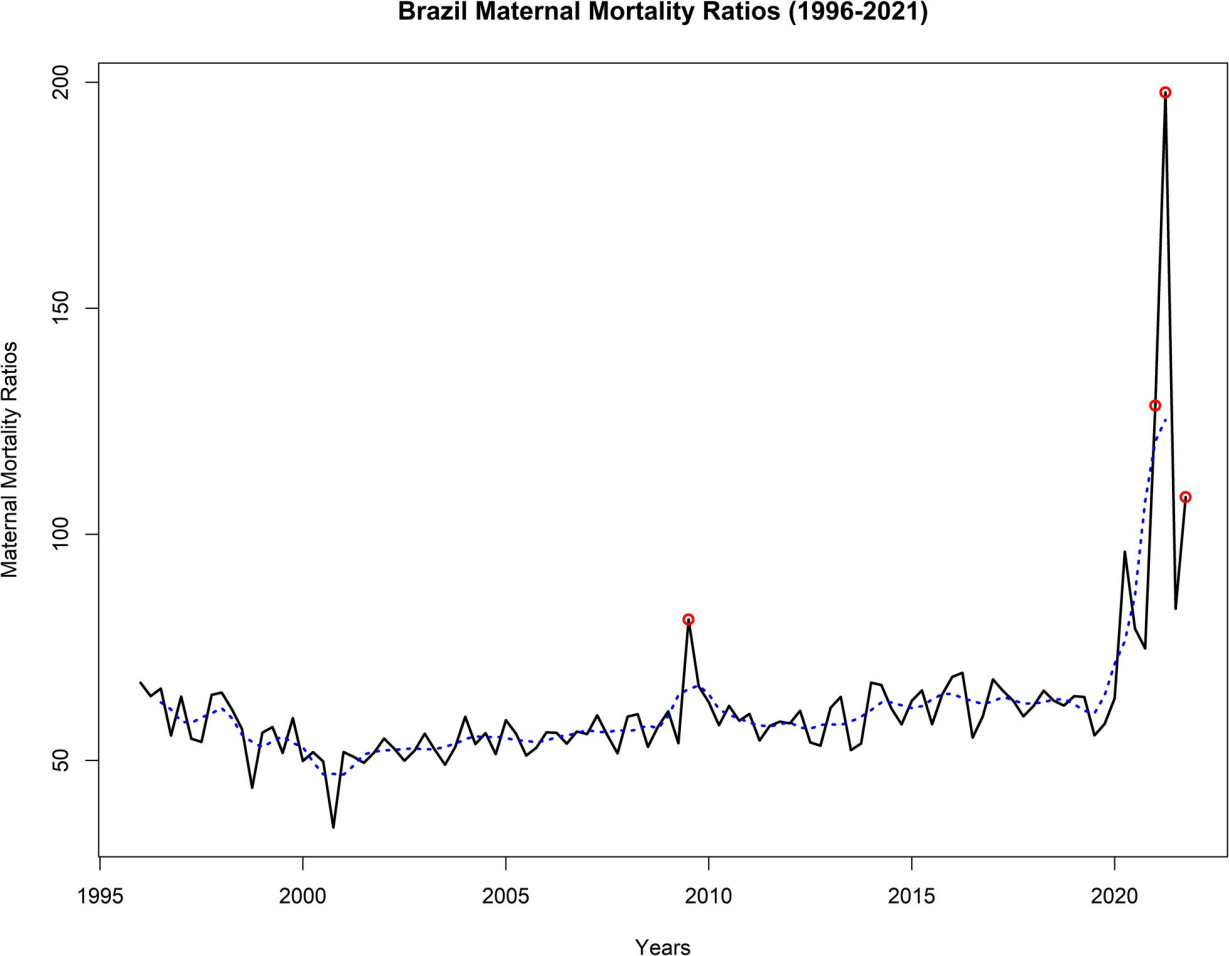
Brazilian Maternal Mortality Ratio (1996–2021), highlighting the H_1_N_1_-influenza (Flu) pandemic (2009) and the COVID-19 pandemic (2020–2021). The blue dash lines are the trend, and the red circle are the outliers.

The BMMR series (Fig 1) reveals that Brazil consistently had high maternal mortality values, considering WHO parameters (mean BMMR [1996–2019] = 57.99 ±6.34/100,000 live births). Notably, during the third quarter of 2009, when analyzing the peak of maternal deaths, the BMMR reached 81.00/100,000 live births, marking a 39.67% increase compared to the mean. This increase was associated with the H1N1 pandemic, which is known to have adverse effects on maternal and perinatal outcomes [12]. Studies have shown that pregnancy and the postpartum period were statistically associated with deaths from pandemic influenza [13].

In August 2020, the Pan American Health Organization (PAHO) highlighted the elevated risk of severe forms of COVID-19 in pregnant women and recommended that countries intensify efforts related to women’s and newborns’ health to ensure access to prenatal and disease prevention services [14]. However, the Brazilian health system faced challenges in identifying cases of pregnant and postpartum women with COVID-19, resulting in delays in the immunization of pregnant and postpartum women, which only began in July 2021 [15]. Consequently, the country experienced the highest mortality ratio in the historical BMMR series during the second quarter of 2021 (169.00/100,000 live births), representing an approximately 191% increase compared to the previous period and a notable contrast with global reports on maternal deaths during the COVID-19 pandemic [2].

Ecological spatial analysis research conducted in Brazil revealed that COVID-19 deaths among pregnant and postpartum women exhibited a heterogeneous geographic distribution, with distinct spatial clusters primarily located in rural areas [16]. Most maternal deaths occurred during the postpartum period, as investigations using data from the Brazilian Ministry of Health’s Epidemiological Surveillance system identified 40,640 women of reproductive age hospitalized for COVID-19. Among them, 3,372 were pregnant, and 794 were postpartum. Postpartum women had a worse prognosis compared to pregnant women, with higher rates of ICU admissions, invasive ventilatory support, and fatalities [17].

A study comparing the first and second waves of COVID-19 in pregnant and postpartum women in Brazil reported 377 maternal deaths in 2020 and 804 in 2021. Furthermore, the COVID-19 mortality rate doubled compared to the previous year. In the obstetric group, the rate increased from 7.7% to 15.4%, while in the non-obstetric group, it increased from 13.9% to 22.9% from 2020 to 2021. Among women with comorbidities, the mortality rate increased by 1.7 times (from 13.3% to 23.3%) and by 1.4 times (from 22.8% to 31.4%) [18]. A retrospective time series study using the Holt-Winters exponential smoothing additive model revealed devastating consequences for maternal mortality during the COVID-19 pandemic in the state of Bahia, Brazil [19].

### Background forecast methods

Several studies have indicated that NNA, Holt-Winters exponential smoothing, and ARIMA are established approaches for forecasting COVID-19 case counts and deaths. For example, Lynch and Gore (2021) assessed seven forecasting methods with varying look-back and forecast lengths at the country, health district, and state levels [20]. Papastefanopoulos *et al*. (2020) employed statistical and machine learning-inspired time series methods to estimate the percentage of active cases relative to the total population in the ten countries with the highest active cases [21]. Barría-Sandoval *et al*. (2021) conducted a comparative analysis of ARIMA models, Exponential Smoothing, State Space models, the Bayesian approach, and the GLARMA model to predict confirmed COVID-19 cases and deaths in Chile [22]. Gecili *et al*. (2021) evaluated four forecast models for predicting COVID-19 confirmed cases, deaths, and recoveries in the USA and Italy based on daily reported data [9].

## Methods

### Data

Maternal death data were obtained from the Mortality Monitoring Panel available on DATASUS (https://svs.aids.gov.br/daent/centrais-de-conteudos/paineis-de-monitoramento/mortalidade/cid10/, accessed on 25/08/2023), filtered by the International Classification of Diseases (ICD-10) using pregnancy, childbirth, and puerperium as indicators. The number of live births was obtained from the Live Births Panel on DATASUS (https://svs.aids.gov.br/daent/centrais-de-conteudos/paineis-de-monitoramento/natalidade/nascidos-vivos/, accessed on 25/08/2023). Data from 1996 to 2021 are consolidated, while data from 2022 onwards are preliminary as of the time of writing.

The BMMR was calculated following the WHO’s International Classification of Diseases. The maternal mortality ratio is defined as the number of maternal deaths per 100,000 live births (Eq 1) [23].


$$\text{Maternal}\;\text{mortality}\;\text{ratio}=\frac{\text{maternal}\;\text{death}}{\text{number}\;\text{of}\;\text{live}\;\text{births}}x\;100,000$$ (1)

The WHO has established parameters to categorize the maternal mortality ratio as low, medium, high, or very high: Low: up to 20/100,000 live births; Average: from 20 to 49/100,000 live births; High: from 50 to 149/100,000 live births; and Very high: > 150/100,000 live births. In this study, the BMMR time series is presented by quarter.

### Study design

We conducted an observational time-series cross-sectional data analysis of quarterly BMMR data. To investigate the impact of COVID-19, we used available pre-pandemic data (2020–2019) to forecast the BMMR up to 2025 (COVID-19-free scenario). Subsequently, we compared the forecast values (2020–2023(1)) with the BMMR reported by the official source. Additionally, we used all historical data (1996–2021) to forecast the BMMR up to 2025 (pandemic scenario). To avoid issues of underfitting or overfitting due to improper training of the dataset, the models underwent cross-validation, with a validation window spanning three years (2016–2019).

#### Package, data set, and reproducibility

All models were constructed using the “forecast” package version 8.15, which incorporates Forecasting Functions for Time Series and Linear Models [10]. All datasets are available in the S1 File. Computer code used for the analyses is provided in the S2 File. All statistical analyses were performed on a computer equipped with two Intel^®^ Xeon^®^ Silver 4214 Processors (13.75 M Cache, 2.20 GHz), 96 GB of RAM, 24 cores, and 64-bit Windows 10 Enterprise, using the RStudio statistical software, Version 1.4.1717.

### Outliers identification

Outliers are observations that significantly differ from the majority of observations in a time series. They may represent errors or unusual events, such as pandemic occurrences. Outliers can alter the dynamics of a time series either temporarily or permanently. These changes are typically non-systematic and cannot be captured by standard time series models. Detecting outliers is crucial because they impact model selection, parameter estimation, and, consequently, forecasts. Outliers were identified using the“tsoutliers()” function in the “forecast” package for R (version 8.15), through an iterative outlier detection and adjustment process using Seasonal Decomposition of Time Series by Loess (with iterate = 2 and lambda = NULL) [24].

### Fitting Holt-Winters model

The HW smoothing technique, also known as triple exponential smoothing, was employed to model the BMMR data. This method offers two variations, differing primarily in their treatment of seasonal components: additive and multiplicative. The choice between these variations depends on the nature of seasonal variations in the data. Additive models are preferable when seasonal variations remain roughly constant throughout the series, while multiplicative models are suitable when seasonal variations change proportionally with the level of the series. In this study, the multiplicative method (Eq 2), the seasonal component is expressed in relative terms (percentages), and the series is seasonally adjusted by dividing by the seasonal component [10]. The multiplicative models provide better results; thus, they were used in this study.

$${\hat{y}}_{t+h(t)}=\left(l_{t}+hb_{t}\right)s_{t+h-m(k+1)}$$ (2)

where *ℓ*_*t*_ represents the level; *b*_*t*_ is the trend; s_*t*_ represents the seasonal component; *m* represents the period of the seasonality (quarterly data), and *h* represents the forecast.

Optimal smoothing weights were computed as follows: α = 0.1571, β* = 0.2434, and γ = 0.1979. The adequacy of the Holt-Winters model was assessed by examining the randomness of the model residuals (S1 Fig). The ACF plot of the residuals showed that all autocorrelations fell within threshold limits, indicating that the residuals behaved like white noise. The histogram of the residuals displayed a symmetrical distribution. Furthermore, the Ljung-Box test (Q* = 5.1668, df = 8, *p*-value = 0.7396) confirmed that the residuals did not significantly deviate from a white noise series.

### Fitting ARIMA model

ARIMA is a statistical analysis model that utilizes time series data to enhance understanding of the dataset or predict future trends. ARIMA models assume a linear correlation between time-series values and aim to use these linear dependencies in observations to extract local patterns while eliminating high-frequency noise from the data [25].

It combines differencing with autoregression and a moving average model, as described by Eq 3.

$$y_{t}^{\prime }=c+\emptyset_{1}y_{t-1}^{\prime }+\cdots +\emptyset_{p}y_{t-p}^{\prime }+\theta_{1}\varepsilon_{t-1}+\cdots +\theta_{q}\varepsilon_{t-q}+\varepsilon_{t}$$ (3)

where $y_{t}^{\prime }$ is the different time series. The “predictors” include both lagged *y*_*t*_ values and errors (*ε*_*t*_). The model is represented by ARIMA (*p*, *d*, *q*), where: ***p*** represents the autoregressive process, the influence of the previous value of the variable on the value under consideration; ***d*** is related to the number of differentiations to induce stationarity; and ***q***, is related to the influence of the noise generated in the previous values [10]. A seasonal ARIMA model is formed by incorporating additional seasonal terms into ARIMA and can be represented as ARIMA (*p*, *d*, *q*) (*P*, *D*, *Q*)_*m*_, where uppercase and lowercase notations denote seasonal and non-seasonal components of the model, and *m* represents the seasonal period.

Although the ARIMA model is valuable and powerful in time series analysis, selecting the appropriate orders for its components can be somewhat challenging. For our purposes focused on the usefulness of the forecast, we chose to determine the orders of the ARIMA model automatically, using the “auto.arima” function in the “forecast” package for R. The function performs a search on the possible model within the constraints of order provided according to the selected metric (such as AIC, AICc, or BIC value). This choice facilitates both the implementation of the model, and its updates as new data becomes available, allowing greater applicability of the model [9].

For optimizing ARIMA models, we employed the Box and Jenkins methodology in a four-step iterative process, as follows: model identification, parameter estimation, diagnostic checking, and prediction [26].

#### 1) Model identification

The ARIMA model is suitable for stationary time series data, as supported by the literature [27, 28]. We verified stationarity using a unit root test, specifically the Kwiatkowski-Phillips-Schmidt-Shin (KPSS) test, calculated using the “urca” package [29]. The obtained *p*-value of 0.1728 for the time series significantly exceeded the 1% critical value, indicating non-stationarity and the need for differencing [29]. After one differencing, the *p*-value became 0.0234, confirming that the differenced data are stationary (S2 Fig).

#### 2) Parameter’s estimation

To determine the structure of correlation between time lags of the differenced data, we assessed autocorrelation function (ACF) and partial autocorrelation (PACF) plots (S2 Fig). After evaluating several models, we selected the ARIMA (0,1,3)(2,0,0) [4] model due to its lowest Akaike’s Information Criteria (AIC) and Bayesian Information Criteria (BIC), making it suitable for fitting the BMMR (1996–2021).

#### 3) Model diagnostics (goodness of fit)

We evaluated the adequacy of the ARIMA (0,1,3)(2,0,0) [4] model by examining the randomness of the residuals (S3 Fig). The ACF plot of the residuals indicated that all autocorrelations were within threshold limits, suggesting that the residuals behaved like white noise. Additionally, the histogram displayed symmetry. The Ljung-Box test (Q* = 3.7432, df = 3, *p*-value = 0.2906) confirmed that the residuals were indistinguishable from a white noise series.

#### 4) Forecasting

We applied the ARIMA (0,1,3)(2,0,0) [4] model to forecast BMMR values up to 2025.

*Fitting NNA model*. NNA is a forecasting method based on mathematical models of the brain, enabling the modeling of complex non-linear relationships between the response variable and its predictors. A key characteristic of NNA is its ability to self-learn without prior knowledge of the intricate non-linear relationships between input and output variables.

In the context of time series data, lagged values of the time series can serve as inputs. The predictors, or inputs, form the bottom layer, while the intermediate layers include “hidden neurons” (optional), and the forecasts or outputs constitute the top layer. In a multilayer feed-forward network, each node layer receives inputs from the previous layers and combines them using weighted linear combinations. For instance, the inputs into hidden neuron *j* are linearly combined, as shown in Eq 4, and this is subsequently modified using a non-linear function like a sigmoid, as depicted in Eq 5.


$$z_{j}=b_{j}+\sum_{i=n}^{n}w_{i,j}x_{i}$$ (4)

$$s\left(z_{j}\right)=\frac{1}{1+e^{-z_{j}}}$$ (5)

Where ***n*** is the number of neurons in the input layer, parameters *b*_*j*_ and *w*_*j*_ are “learned” (or estimated) from the data during the training of the model. The notation NNA (*p*, *k*, *n*) indicates there are *p*-lagged inputs and *k* nodes in the hidden layer [10].

Various parameters were tested and evaluated (S1 Table). Considering all combinations of these parameters, 2,400 different NNA models were trained. Among these models, the NNA (8,6,1) [6] demonstrated superior performance by yielding the lowest Root Mean Squared Error (RMSE) [30]. In this particular model, the last eight observations are utilized as non-seasonal predictors, six as seasonal lag predictors, and one neuron is placed in the hidden layer.

The suitability of the NNA (8,6,1) [6] model was assessed based on the randomness of model residuals (S4 Fig). The ACF plot of the residuals indicates that all autocorrelations fall within the threshold limits, suggesting that the residuals exhibit behavior similar to white noise. Additionally, the histogram displays symmetry. The Ljung-Box test (Q* = 6.6877, df = 8, *p*-value = 0.5707) confirms that the residuals cannot be distinguished from a white noise series.

*Models’ comparison*. When comparing forecast methods applied to a single time series or to multiple time series with the same units, the mean absolute error (MAE, Eq 6) is the most commonly used metric because it is easy to understand and compute. On the other hand, the root mean squared error (RMSE, Eq 7) minimizes the impact of bias and measures the dispersion of prediction errors, indicating model stability, despite being somewhat challenging to interpret [10]. A forecasting method that minimizes MAE tends to produce forecasts closer to the median, while minimizing RMSE results in forecasts closer to the mean. RMSE and MAE were employed as performance indicators for comparing the fitted models:

$$MAE=\sum_{i=1}^{n}\frac{\left|{\hat{y}}_{l}-y_{i}\right|}{n}$$ (6)

$$RMSE=\sqrt{\sum_{i=1}^{n}\frac{{\left(\hat{y_{l}}-y_{i}\right)}^{2}}{n}}$$ (7)

Additionally, p-values were computed using a two-sample Student’s t-Test on data sets from two independent populations (reported *versus* predicted) with unequal variances. H_0_: true difference between reported and forecast BMMR is equal to zero (significance level of ɑ = 0.02).

### Ethical aspects

All data utilized in this study were obtained from publicly available sources through the Office of Public Health. Consequently, the study did not undergo ethical review.

## Results and discussion

### Pandemic events

The sudden increase in BMMR during the second quarter of 2009 (81.00/100,000 live births) is associated with the H1N1-Influenza (Flu) pandemic, while the peak (197.75/100,000 live births) in the second quarter of 2021 is related to the COVID-19 pandemic (2020–2021). Predicting the occurrence of the next pandemic is unfeasible due to the random nature of such events. Therefore, for modeling purposes, these events were treated as outliers [31]. Four outliers were identified in the BMMR time series for the years 1996 to 2021 (Fig 1).

### Holt-Winters, ARIMA, and NNA forecasts

**Fig 2:**
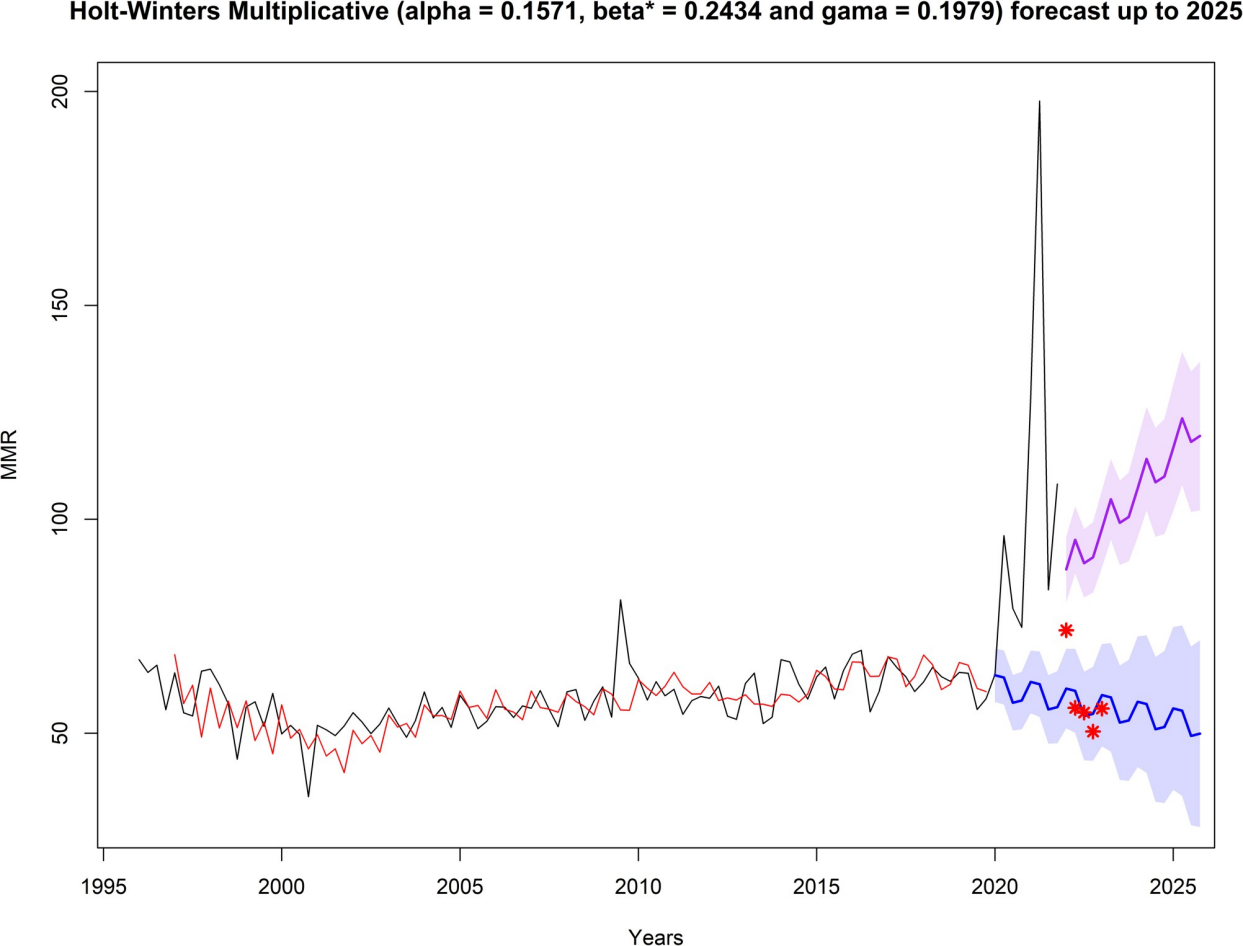
Forecast of the quarterly Brazil Maternal Mortality Ratio up to 2025 using the Holt-Winters multiplicative method. : Black line represents the quarterly Brazil Maternal Mortality Ratio (1996–2021) reported by the official source; red line represents values provided by the Holt-Winters model; blue line represents forecast values in a controlled COVID-19 scenario; purple line represents forecast values in a pandemic COVID-19 scenario, and the shaded area indicates the 80% confidence interval limits.

The Holt-Winters forecasts for controlled and pandemic COVID-19 scenarios are presented in **Fig 2**. In a controlled COVID-19 scenario, the BMMR exhibits a decreasing trend, reaching a ratio of 49.90/100,000 live births, with an 80% confidence interval (CI) of [28.07, 71.74] in 2025. However, in a pandemic scenario, the BMMR shows an increasing trend, reaching a ratio of 119.47/100,000 live births, with an 80% CI of [102.15, 136.80] in 2025.

**Fig 3:**
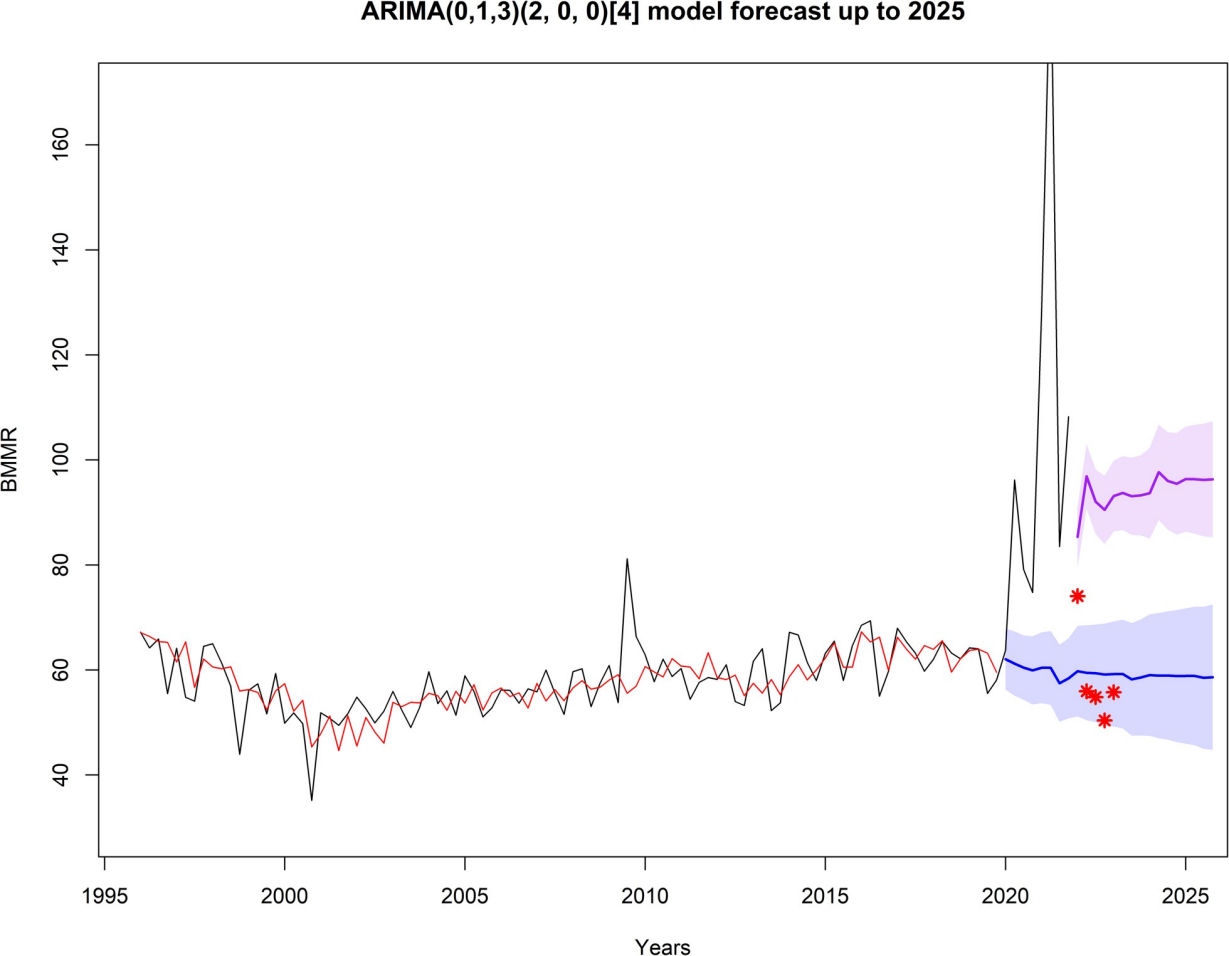
Forecast of the quarterly Brazil Maternal Mortality Ratio up to 2025 using ARIMA (0,1,3) (2, 0, 0) [4]. The black line represents the quarterly Brazil Maternal Mortality Ratio (1996–2021) reported by the official source; the red line represents values provided by the ARIMA model; the blue line represents forecast values in a controlled COVID-19 scenario; the purple line represents forecast values in a pandemic COVID-19 scenario, and the shaded area indicates the 80% confidence interval limits.

The ARIMA forecast for controlled and pandemic COVID-19 scenarios is presented in **Fig 3**. In a controlled COVID-19 scenario, the BMMR exhibits a stable trend, reaching a ratio of 58.62/100,000 live births, with an 80% CI of [44.78, 72.46] in 2025. However, in a pandemic scenario, the BMMR shows an increasing trend, reaching a ratio of 96.29/100,000 live births, with an 80% CI of [85.25, 107.34] in 2025.

**Fig 4:**
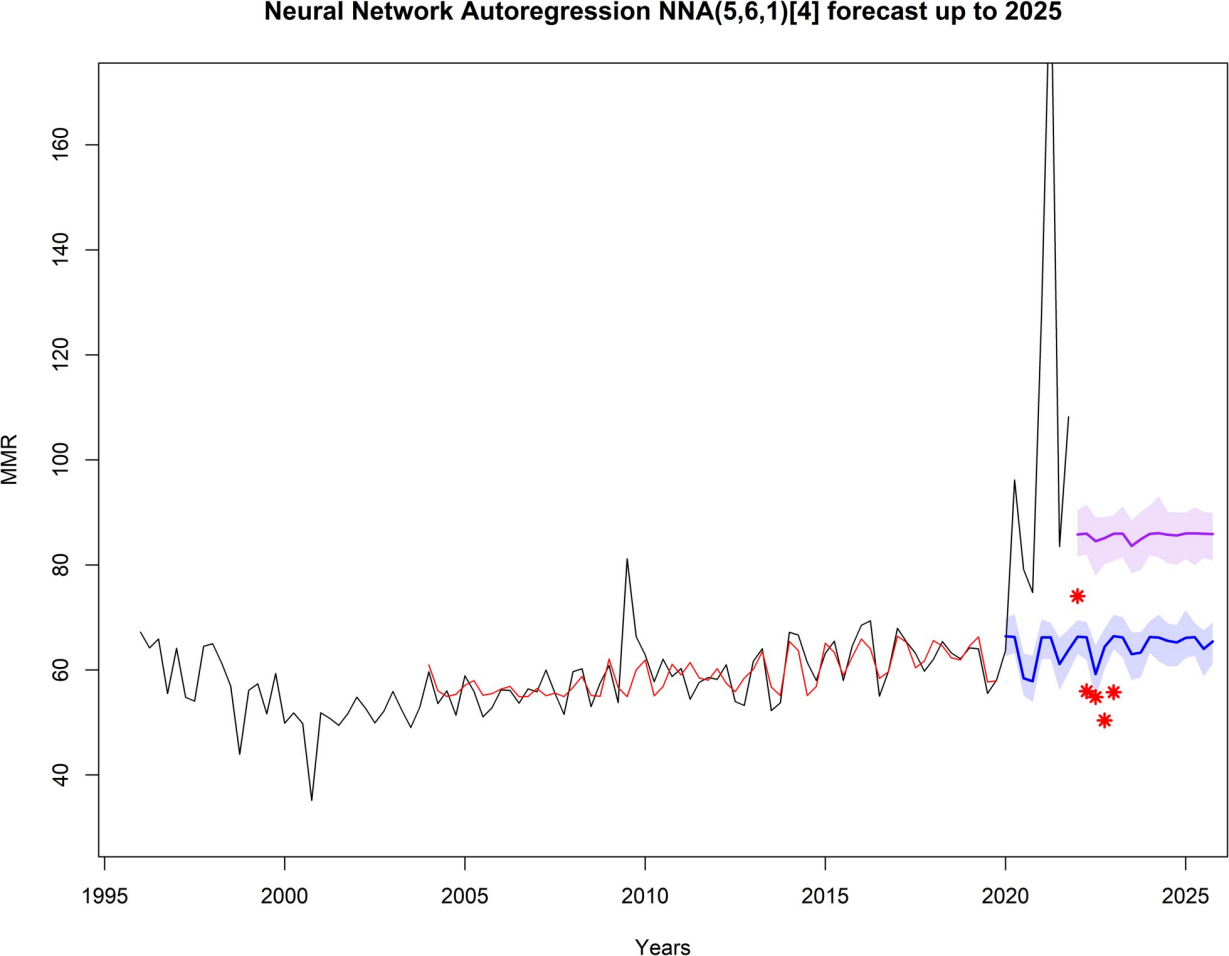
Forecast of the quarterly Brazil Maternal Mortality Ratio up to 2025 using the NNA (8,6,1) [6] model. The black line represents the quarterly Brazil Maternal Mortality Ratio (1996–2021) reported by the official source; the red line represents values provided by the NNA model; the blue line represents forecast values in a controlled COVID-19 scenario; the purple line represents forecast values in a pandemic COVID-19 scenario, and the shaded area indicates the 80% confidence interval limits.

The NNA forecast for controlled and pandemic COVID-19 scenarios is presented in **Fig 4**. In a controlled COVID-19 scenario, the BMMR exhibits a stable trend, reaching a ratio of 65.47/100,000 live births, with an 80% CI of [61.08, 68.96] in 2025. However, in a pandemic scenario, the BMMR shows a slightly increasing trend, reaching a ratio of 85.60/100,000 live births, with an 80% CI of [81.10, 92.75] in 2025.

#### Models performance

The performance of the models was evaluated based on a test set with twelve samples. The RMSE for NNA (4.5701/100,000 live births) was lower than that for Holt-Winters (7.1026/100,000 live births) and ARIMA (4.7613/100,000 live births). In contrast, the MAE was the lowest for ARIMA (3.6750/100,000 live births), compared to Holt-Winters (6.0426/100,000 live births) and NNA (3.7579/100,000 live births). Additionally, NNA provided a small value for Cross-Validation (CV) and a better-adjusted R^2^, while ARIMA provided small values for Akaike’s Information Criterion (AIC) and Schwarz’s Bayesian Information Criterion (BIC), as shown in Table 1. The ARIMA and NNA models appear to be more suitable at this stage.

**Table 1:** Performance parameters of evaluated models.

	Holt-Winters	ARIMA	NNA
RMSE*	7.102567	4.761288	4.570186
MAE*	6.042601	3.674994	3.757938
CV	18.85876	13.23995	11.86506
AIC	225.1274	208.6428	278.1728
BIC	232.1196	215.7889	370.7265
AdjR^2^	0.309856	0.427773	0.799221
*p*-value**	0.000391	0.031790	0.135500

*Expressed as number of deaths per 1,000 individuals, per year

**H_0_: true difference between reported and forecast BMMR is equal to zero (significance level of ɑ = 0.02).

### Increase in mortality associated with COVID-19

The BMMR reported during the COVID-19 pandemic (2020–2021) was significantly higher than previous (1996–2019; p < 0.01) and forecasted (2022–2025; p < 0.01) values, as shown in Table 2. This underscores the impact of the pandemic on maternal mortality. Notably, the BMMR returned to historical levels after the second quarter of 2022, coinciding with the Ministry of Health of Brazil declaring the end of the Public Health Emergency of National Importance due to Covid-19 on April 22, 2022. Considering the forecast results beyond the sample period (2022 and beyond), the Holt-Winters model demonstrated its superiority in forecasting the BMMR time series. Although ARIMA and NNA models provided an excellent fit to the historical data, the Holt-Winters model exhibited better alignment with the preliminary data reported in 2022 and the first quarter of 2023. Additionally, it is worth noting that the confidence intervals obtained for Holt-Winters and ARIMA included the reported values.

**Table 2:** Quarterly forecast of the Brazilian Maternal Mortality Ratio (out of 1,000 live births) up to 2025 (in a controlled COVID-19 scenario), comparing forecasted values with reported values (2020–2021).

	Reported	Holt-Winters			ARIMA			NNA		
Year(quarter)		Mean	[80% CI]		Mean	[80% CI]		Mean	[80% CI]	
2020(1)	63.74	63.55	[44.78	67.85]	62.06	[44.78	67.85]	66.50	[61.08	70.03]
2020(2)	*96*.*17*	63.02	[44.97	67.33]	61.21	[44.97	67.33]	66.34	[59.69	69.50]
2020(3)	*79*.*15*	57.12	[45.65	66.58]	60.45	[45.65	66.58]	58.41	[61.93	63.61]
2020(4)	*74*.*76*	57.65	[45.96	66.41]	59.92	[45.96	66.41]	57.80	[61.60	61.71]
2021(1)	*128*.*47*	62.00	[46.27	67.15]	60.42	[46.27	67.15]	66.27	[59.79	68.63]
2021(2)	*197*.*75*	61.47	[46.72	67.43]	60.40	[46.72	67.43]	66.30	[60.70	70.09]
2021(3)	*83*.*52*	55.57	[47.00	64.81]	57.47	[47.00	64.81]	61.09	[62.60	65.82]
2021(4)	*108*.*23*	56.10	[47.47	66.00]	58.39	[47.47	66.00]	63.78	[61.76	68.54]
2022(1)	*74*.06*	60.45	[47.51	68.38]	59.76	[47.51	68.38]	66.42	[59.43	70.48]
2022(2)	55.94*	59.92	[47.50	68.50]	59.47	[47.50	68.50]	66.29	[57.44	69.91]
2022(3)	54.83*	54.02	[48.88	68.64]	59.37	[48.88	68.64]	59.10	[62.35	64.53]
2022(4)	50.39*	54.55	[49.22	68.81]	59.13	[49.22	68.81]	64.39	[62.18	68.30]
2023(1)	55.76*	58.90	[49.45	69.23]	59.22	[49.45	69.23]	66.53	[59.01	70.10]
2023(2)		58.37	[50.09	69.59]	59.23	[50.09	69.59]	66.28	[54.40	69.90]
2023(3)		52.48	[50.43	68.93]	58.21	[50.43	68.93]	62.96	[62.24	67.95]
2023(4)		53.00	[51.14	69.59]	58.55	[51.14	69.59	63.25	[63.21	67.49]
2024(1)		57.35	[50.78	70.58]	59.03	[50.78	70.58]	66.35	[58.62	70.26]
2024(2)		56.83	[50.14	70.84]	58.92	[50.14	70.84]	66.24	[56.24	69.11]
2024(3)		50.93	[53.37	71.17]	58.95	[53.37	71.17]	65.57	[62.90	69.60]
2024(4)		51.45	[53.69	71.42]	58.84	[53.69	71.42]	65.28	[62.18	69.48]
2025(1)		55.80	[53.43	71.73]	58.85	[53.43	71.73]	66.17	[54.07	70.08]
2025(2)		55.28	[54.32	72.07]	58.86	[54.32	72.07]	66.32	[55.12	69.73]
2025(3)		49.38	[55.09	72.03]	58.50	[55.09	72.03]	64.02	[62.76	68.51]
2025(4)		49.90	[56.27	72.46]	58.62	[56.27	72.46]	65.47	[62.50	68.96]
p-value		0.110			0.027			0.017		

*The 2022 and 2023(1) BMMR data are preliminary and may change during review process. These data were not used in the forecast models, and were included during the writing process, only to demonstrate the efficiency of the models. **p-value**: calculated by two-sample student’s t-test on data sets from two independent populations (reported *versus* predicted [2022(2)–2023(1)]) with unequal variances. H_0_: true difference between reported and forecast BMMR is equal to zero (significance level of ɑ = 0.02).

The study compared the forecasting capabilities of artificial intelligence technology and traditional statistical methods for predicting BMMR up to 2025. The use of a long 25-year time series dataset enhances the stability of the models. For the in-sample forecast period of 1996–2021 and the out-of-sample forecast period of 2022–2025, Holt-Winter and ARIMA models demonstrated better alignment with the prediction results beyond the sample and the values reported after the end of the COVID-19 pandemic. However, NNA provided a better fit to the historical data. Papastefanopoulos et al. (2021) found that traditional statistical methods, such as ARIMA and TBAT, generally outperformed deep learning methods, like DeepAR and N-BEATS, in estimating active COVID-19 cases for ten countries [21].

The results indicate that even if the COVID-19 pandemic stabilizes, Brazil is unlikely to achieve the maternal mortality target outlined in the SDGs in the coming years. Therefore, forecast studies play a crucial role in informing more effective actions for preventing maternal deaths. Improvements in living conditions, access to quality healthcare, and comprehensive care are essential to preventing maternal deaths related to pregnancy, childbirth, and the postpartum period. The historical data also highlight that events such as H1N1 and COVID-19 expose existing weaknesses in maternal and child health in the country.

It is important to note that accurate data preprocessing for time series models can be challenging, often requiring a trial-and-error approach. Additionally, NNA methods are computationally intensive and demand powerful computing resources and time. Lastly, a limitation of the study is the use of secondary data published in Brazil, and the data on maternal deaths in 2022 being preliminary.

#### Limitations

Threats to internal validity relate to factors that affect the dependent variables without the researchers’ knowledge [20]. It is possible that some implementation flaws affected subsequent modeling or data analysis. To minimize this risk, the algorithms in our source code were (1) built in established libraries, (2) underwent internal reviews, and (3) are publicly accessible, along with the data and results. Threats to external validity pertain to the generalizability of the analysis results. The results obtained are specific to Brazil, limited to BMMR reported by DATASUS (official database), and based on preliminary data for 2022. These results may not be immediately applicable to (1) different causes of death, (2) other datasets, (3) different time periods, or (4) different geographic regions. Additionally, the selected forecasting methods assume that public health policies remain unchanged during the forecast periods. To enhance model performance, forecast analyses should be regularly updated as new data becomes available, thereby improving the accuracy of these models.

## Conclusion

This study highlights the increase in BMMR and its temporal association with the incidence of Influenza A H1N1 and the COVID-19 pandemic in Brazil. Moreover, it demonstrates that ARIMA and Holt-Winters models were the most effective forecasting methods for modeling maternal mortality when compared to neural networks. These forecasts contribute to the formulation of more effective public policies for the maternal and child population and the expansion of access to healthcare services. Furthermore, these models are readily accessible, as they are implemented in an R package, and can be easily applied by analysts. The fitting process for each model only takes a few minutes, making them conducive to regular updates as new data becomes available.

## Supporting information

S1 File Historical data of BMMR from 1996–2022. (CSV)

S2 File Computer code used for the analyses. (TXT)

S1 Fig Diagnostic plots for Holt-Winters model on Brazilian Maternal Mortality Ratios (1996–2019): (A) residual plot; (B) partial autocorrelation function plot of residual and (C) histogram of residual.
(TIF)

S2 Fig Adequacy of the ARIMA model by (A) Brazilian Maternal Mortality Ratios (1996–2019); (B) first order difference; (C) autocorrelation function plot and (D) partial autocorrelation function.
(TIF)

S3 Fig Diagnostic plots for ARIMA (0,1,3) (2, 0, 0)[4] on Brazilian Maternal Mortality Ratios (1996–2019): (A) residual plot; (B) partial autocorrelation function plot of residual and (C) histogram of residual. (TIF)

S4 Fig Diagnostic plots for NNA (8,6,1)[6] on Brazilian Maternal Mortality Ratios (1996–2019): (A) residual plot; (B) partial autocorrelation function plot of residual and (C) histogram of residual
(TIF)

S1 Table NNA parameters evaluated to BMMR forecast. (DOCX)

## References

[1] Nakamura-Pereira M, Amorim MMR, Pacagnella RC, Libertad M, Takemoto S, Cristina F, et al. COVID-19 e morte materna no Brasil: uma tragédia invisível. FEMINA. 2020;48(6):496–504.

[2] Takemoto MLS, Menezes MO, Andreucci CB, Knobel R, Sousa L, Katz L, et al. Clinical characteristics and risk factors for mortality in obstetric patients with severe COVID-19 in Brazil: a surveillance database analysis. BJOG. 2020;127(13):1618–1626. doi: 10.1111/1471-0528.16470 32799381 PMC7461482

[3] Castro R. Observatório Covid-19 destaca alta mortalidade materna. 2021. Available: https://portal.fiocruz.br/noticia/observatorio-covid-19-destaca-alta-mortalidade-materna. Accessed 24 Jul 2022.

[4] Souza ASR, Amorim MMR. Maternal mortality by COVID-19 in Brazil. Revista Brasileira de Saúde Materno Infantil. 2021;21(1):253–256. doi: 10.1590/1806-9304202100S100014

[5] Ramazani IBE, Ntela SDM, Ahouah M, Ishoso DK, Monique RT. Maternal mortality study in the Eastern Democratic Republic of the Congo. BMC Pregnancy Childbirth. 2022;22:452–466. doi: 10.1186/s12884-022-04783-z 35641954 PMC9153209

[6] Gonçalves BMM, Franco RPV, Rodrigues AS. Maternal mortality associated with COVID-19 in Brazil in 2020 and 2021: Comparison with non-pregnant women and men. PLoS ONE. 2021;16(12):e0261492. doi: 10.1371/journal.pone.0261492 34932589 PMC8691656

[7] Takemoto MLS, Menezes MO, Andreucci CB, Nakamura-Pereira M, Amorim MMR, Katz L, et al. The tragedy of COVID-19 in Brazil: 124 maternal deaths and counting. Int J Gynaecol Obstet. 2020;151(1):154–156. doi: 10.1002/ijgo.13300 32644220 PMC9087660

[8] WHO. World Health Organization. Global health sector strategy on Sexually Transmitted Infections, 2016–2021. 2016 [cited 24 Jul 2022]. Available: https://www.who.int/publications/i/item/WHO-RHR-16.09

[9] Gecili E, Ziady A, Szczesniak RD. Forecasting COVID-19 confirmed cases, deaths and recoveries: Revisiting established time series modeling through novel applications for the USA and Italy. PLoS ONE. 2021;16(1):e0244173. doi: 10.1371/journal.pone.0244173 33411744 PMC7790225

[10] Hyndman R, Athanasopoulos G. Forecasting: Principles and Practice (3rd ed). Melbourne, Australia; 2021. Available: https://otexts.com/fpp3/

[11] Fu J, Cai W, Zeng B, He L, Bao L, Lin Z, et al. Development and validation of a predictive model for peripherally inserted central catheter-related thrombosis in breast cancer patients based on artificial neural network: A prospective cohort study. Int J Nurs Stud. 2022;135:104341. doi: 10.1016/j.ijnurstu.2022.104341 36084529

[12] Al-Husban N, Obeidat N, Al-Kuran O, Oweidat KA, Bakri F. H1N1 infection in pregnancy; a retrospective study of feto-maternal outcome and impact of the timing of antiviral therapy. Mediterr J Hematol Infect Dis. 2019;11(1):e2019020. doi: 10.4084/MJHID.2019.020 30858958 PMC6402546

[13] Pastorello CM, Rochembach A, Doring M, Moretto EFS, Petuco VM, Dalmolin BM, et al. Impacto da influenza pandêmica (H1N1) 2009 e de doenças respiratórias na mortalidade de mulheres em idade fértil no estado do Rio Grande do Sul, Brasil, 2008–2009. Epidemiol e Serv Saúde. 2012;21(2):205–212. doi: 10.5123/S1679-49742012000200003

[14] OPS/OMS. Organización Panamericana de la Salud. Alerta Epidemiológica COVID-19 durante el embarazo. 2019 [cited 24 Jul 2022]. Available: https://pesquisa.bvsalud.org/portal/resource/pt/biblio-1117103

[15] Andreucci CB, Knobel R. Social determinants of COVID-19-related maternal deaths in Brazil]. Lancet Reg Health Am. 2021;3:100104. doi: 10.1016/j.lana.2021.100104 35005689 PMC8720373

[16] Siqueira TS, Silva JRS, Souza MR, Leite DCF, Edwards T, Martins-Filho PR, et al. Spatial clusters, social determinants of health and risk of maternal mortality by COVID-19 in Brazil: a national population-based ecological study. Lancet Reg Health Am. 2021;3:100076. doi: 10.1016/j.lana.2021.100076 34541570 PMC8432892

[17] Serra FE, Francisco RPV, Rossi P, Brizot ML, Rodrigues AS. COVID-19 outcomes in hospitalized puerperal, pregnant, and neither pregnant nor puerperal women. PLoS ONE. 2021;16(11):e0259911. doi: 10.1371/journal.pone.0259911 34780549 PMC8592461

[18] Scheler CA, Discacciati MG, Vale DB, Lajos GJ, Surita FG, Teixeira JC. Maternal Deaths from COVID-19 in Brazil: Increase during the Second Wave of the Pandemic. Rev Bras Ginecol Obstet. 2022;44(6):567–572. doi: 10.1055/S-0042-1748975 35649424 PMC9948055

[19] Carvalho-Sauer RCO, Costa MCN, Teixeira MG, Nascimento EMR, Silva EMF, Barbosa MLA, et al. Impact of COVID-19 pandemic on time series of maternal mortality ratio in Bahia, Brazil: analysis of period 2011–2020. BMC Pregnancy Childbirth. 2021;21:1–7. doi: 10.1186/S12884-021-03899-Y 34112099 PMC8190975

[20] Lynch CJ, Gore R. Short-Range Forecasting of COVID-19 During Early Onset at County, Health District, and State Geographic Levels Using Seven Methods: Comparative Forecasting Study. J Med Internet Res. 2021;23(3):e24925. doi: 10.2196/24925 33621186 PMC7990039

[21] Papastefanopoulos V, Linardatos P, Kotsiantis S. COVID-19: A Comparison of Time Series Methods to Forecast Percentage of Active Cases per Population. Appl. Sci. 2020; 10(11):3880. doi: 10.3390/app10113880

[22] Barría-Sandoval C, Ferreira G, Benz-Parra K, Ló Pez-Flores P. Prediction of confirmed cases of and deaths caused by COVID-19 in Chile through time series techniques: A comparative study. PLoS ONE. 2021;16(4):e0245414. doi: 10.1371/journal.pone.0245414 33914758 PMC8084230

[23] WHO. World Health Organization. Classificação Estatística Internacional de Doenças e Problemas Relacionados à Saúde. 10th ed. 2017. [cited 24 Jul 2023]. Available: https://www.edusp.com.br/loja/produto/74/cid10-vol—1—classificacao-estatistica-internacional-de-doencas-e-problemas-relacionados-a-saude

[24] Hyndman (2021). “Detecting time series outliers”. [cited 24 Jul 2023]. Available: https://robjhyndman.com/hyndsight/tsoutliers/.

[25] Adhikari R, Agrawal RK. An Introductory Study on Time Series Modeling and Forecasting. LAP. 2013. doi: 10.48550/arXiv.1302.6613

[26] Adeyinka DA, Muhajarine N. Time series prediction of under-five mortality rates for Nigeria: comparative analysis of artificial neural networks, Holt-Winters exponential smoothing and autoregressive integrated moving average models. BMC Med Res Methodol. 2020;20:1–11. doi: 10.1186/s12874-020-01159-9 33267817 PMC7712624

[27] Lynch CJ, Gore R. Application of one-, three-, and seven-day forecasts during early onset on the COVID-19 epidemic dataset using moving average, autoregressive, autoregressive moving average, autoregressive integrated moving average, and naïve forecasting methods. Data Brief. 2021;35:106759. doi: 10.1016/j.dib.2021.106759 33521186 PMC7834853

[28] Castle JL, Doornik JA, Hendry DF. The value of robust statistical forecasts in the Covid-19 pandemic. Natl Inst Econ Ver. 2021; 256:19–43. doi: 10.1017/nie.2021.9

[29] Kwiatkowski D, Phillips PCB, Schmidt P, Shin Y. Testing the null hypothesis of stationarity against the alternative of a unit root: How sure are we that economic time series have a unit root? J Econom. 1992;54:159–178. doi: 10.1016/0304-4076(92)90104-Y

[30] Chowell G. Fitting dynamic models to epidemic outbreaks with quantified uncertainty: A primer for parameter uncertainty, identifiability, and forecasts. Infect Dis Model. 2017;2(3):379–398. doi: 10.1016/j.idm.2017.08.001 29250607 PMC5726591

[31] Heymnna D, Ross E, Wallece J. The next pandemic–when could it be? 23 Feb 2022 [cited 24 Jul 2022]. Available: https://www.chathamhouse.org/2022/02/next-pandemic-when-could-it-be

