# Supplementary figures and images for: Impact of COVID-19 pandemic in the Brazilian maternal mortality ratio: A comparative analysis of Neural Networks Autoregression, Holt-Winters exponential smoothing, and Autoregressive Integrated Moving Average models
Source: PLoS One. 2024 Jan 31;19(1):e0296064. doi: 10.1371/journal.pone.0296064 (PMC10830046; doi:10.1371/journal.pone.0296064)

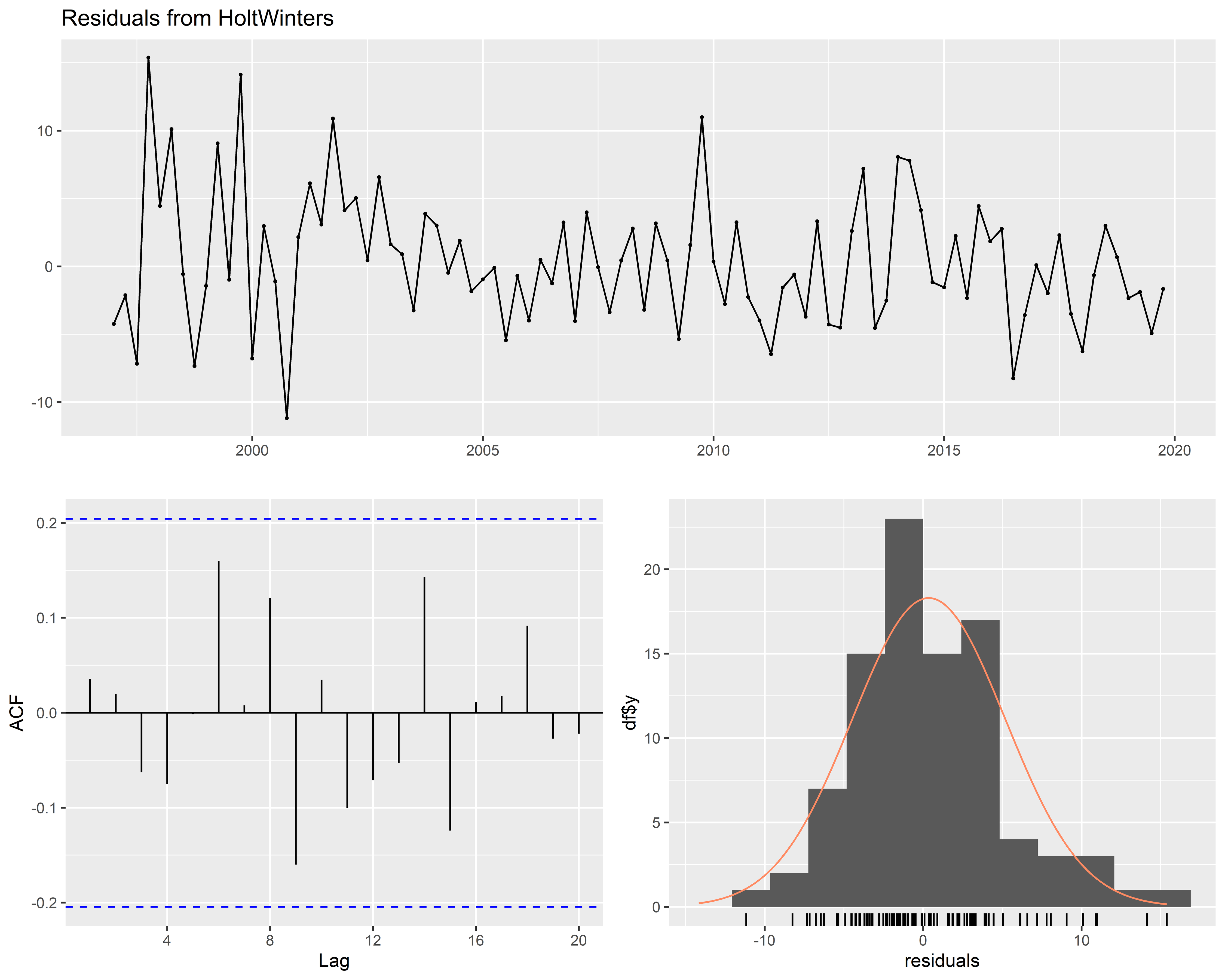

Supplement: S1 Fig — Diagnostic plots for Holt-Winters model on Brazilian Maternal Mortality Ratios (1996–2019): (A) residual plot; (B) partial autocorrelation function plot of residual and (C) histogram of residual. (TIF) [file pone.0296064.s003.tif]

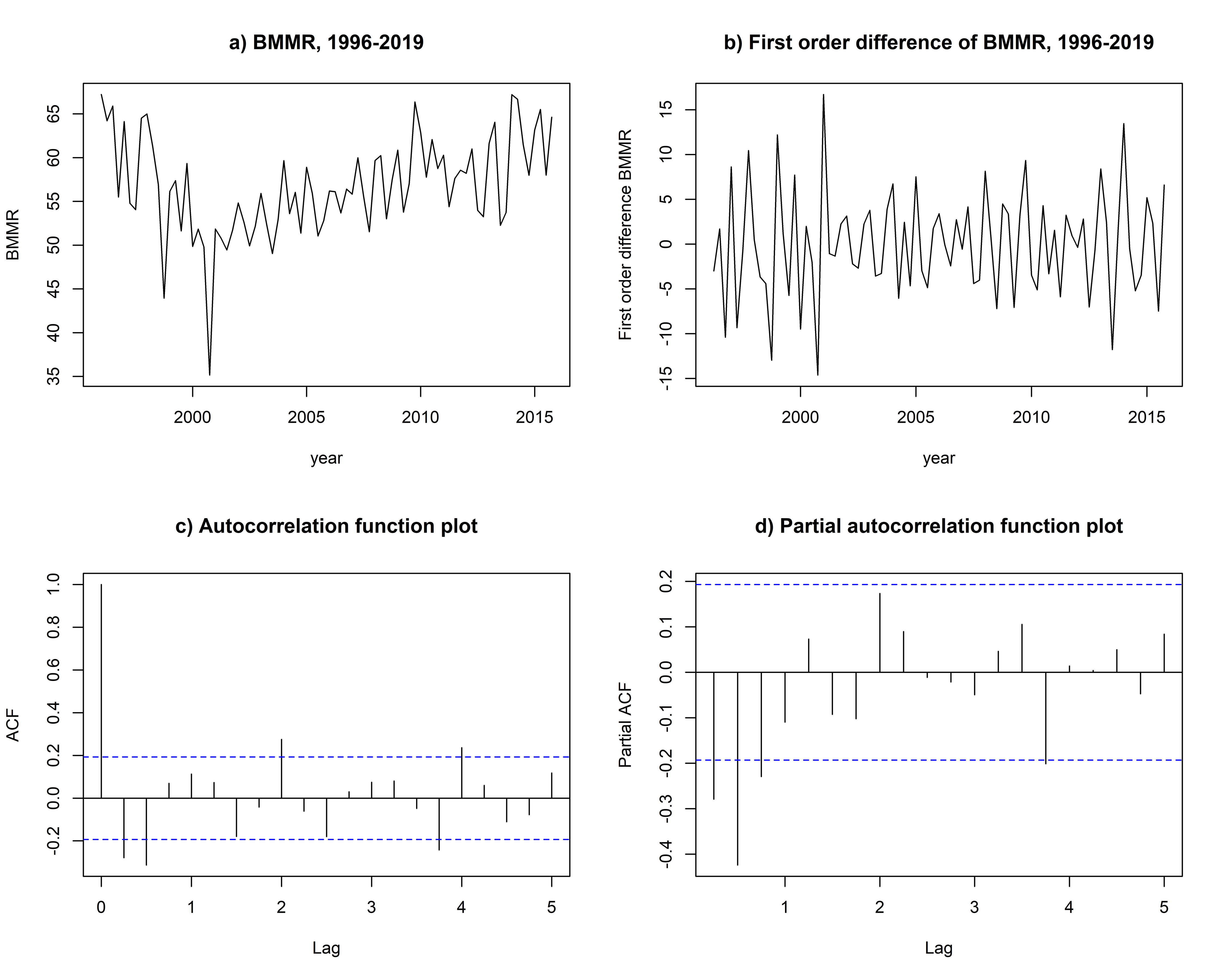

Supplement: S2 Fig — Adequacy of the ARIMA model by (A) Brazilian Maternal Mortality Ratios (1996–2019); (B) first order difference; (C) autocorrelation function plot and (D) partial autocorrelation function. (TIF) [file pone.0296064.s004.tif]

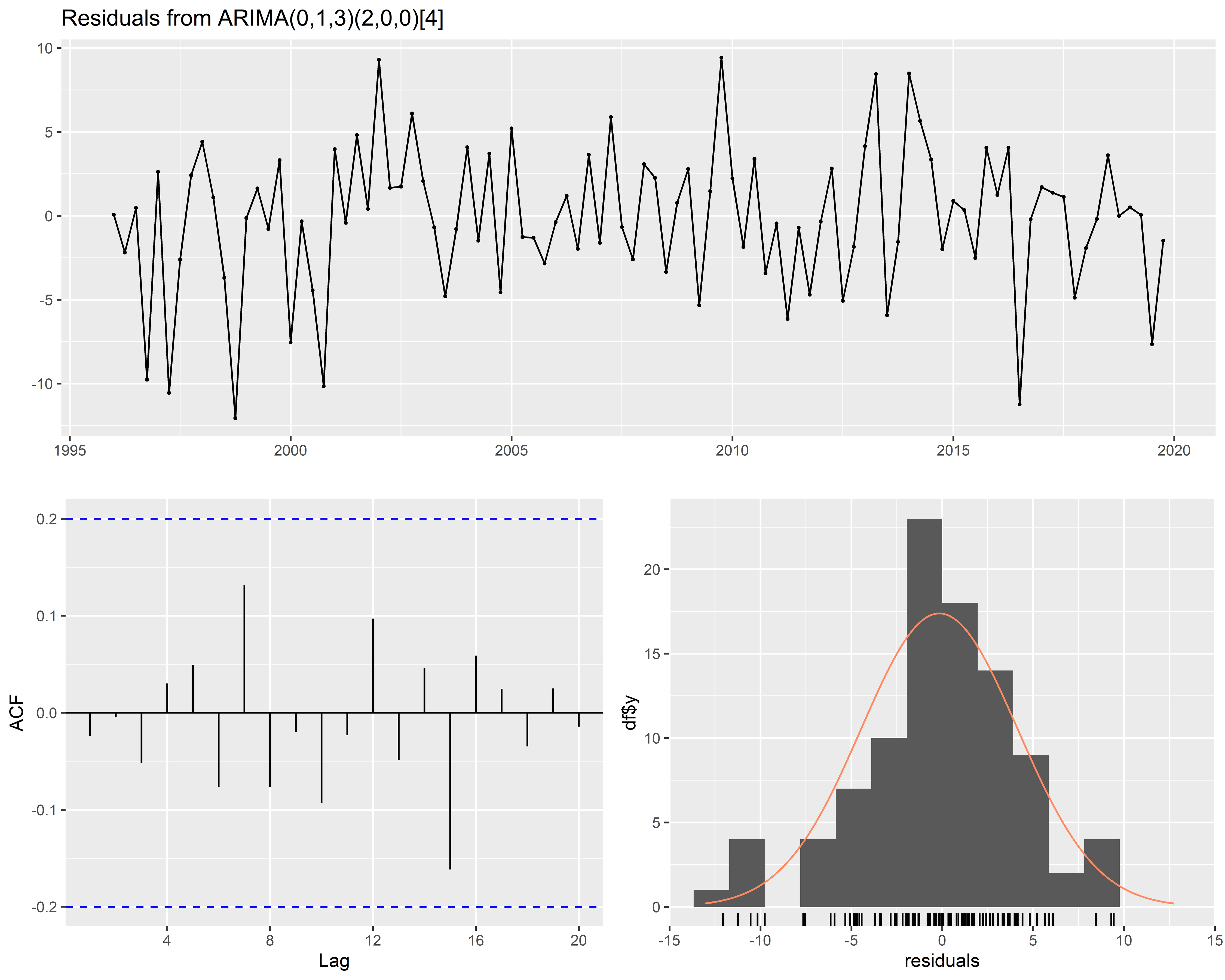

Supplement: S3 Fig — (TIF) [file pone.0296064.s005.tif]

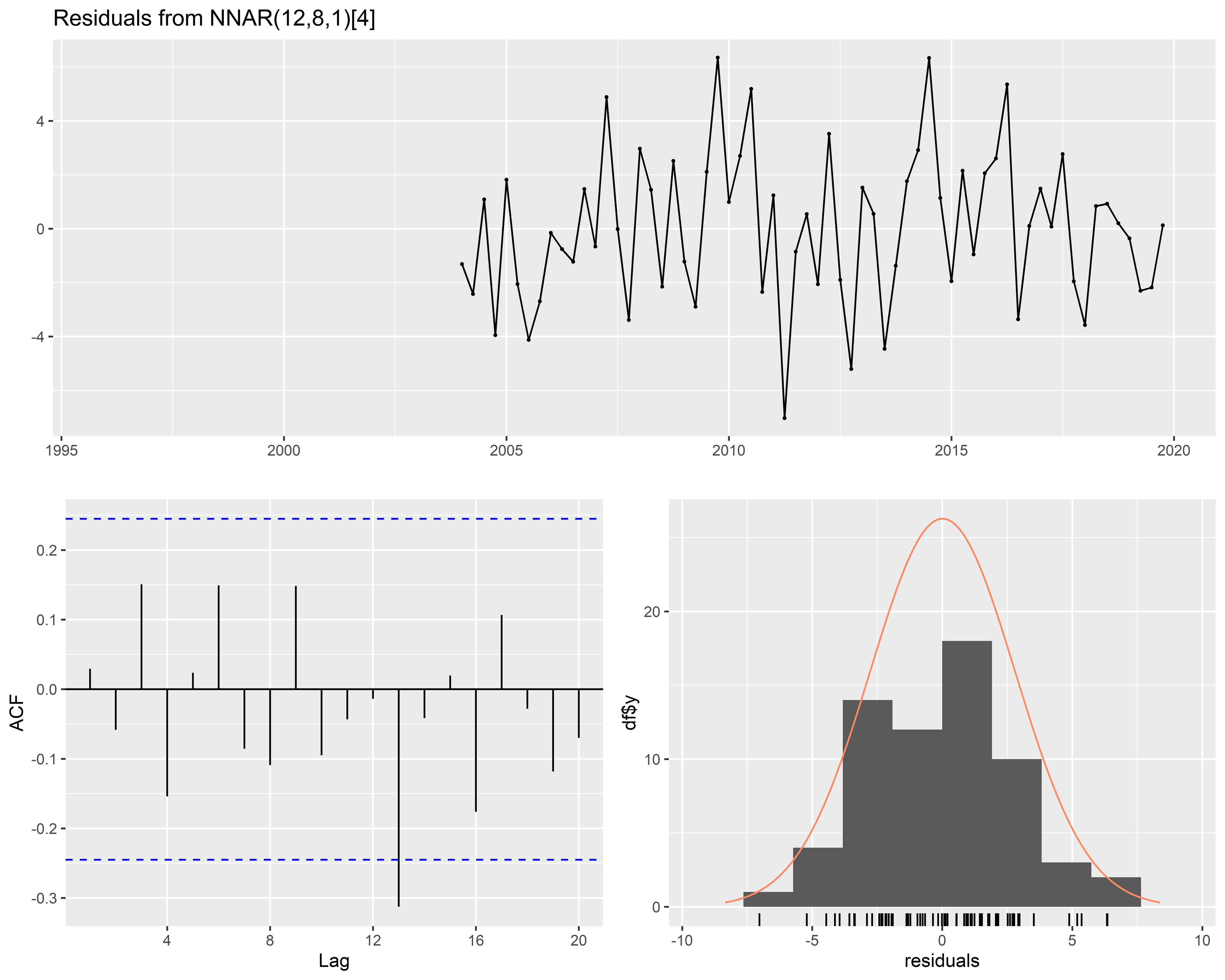

Supplement: S4 Fig — Diagnostic plots for NNA (8,6,1)[6] on Brazilian Maternal Mortality Ratios (1996–2019): (A) residual plot; (B) partial autocorrelation function plot of residual and (C) histogram of residual (TIF) [file pone.0296064.s006.tif]
