# Supplementary material for: Impact of COVID-19 pandemic in the Brazilian maternal mortality ratio: A comparative analysis of Neural Networks Autoregression, Holt-Winters exponential smoothing, and Autoregressive Integrated Moving Average models
Source: PLoS One. 2024 Jan 31;19(1):e0296064. doi: 10.1371/journal.pone.0296064 (PMC10830046; doi:10.1371/journal.pone.0296064)
Supplement: S1 Table — (DOCX) [file pone.0296064.s007.docx]

**Table S1** - NNA Parameters evaluation to BMMR forecast.

| **#** | **NNA Parameters** | **Evaluated values** | **Best parameters** |
| --- | --- | --- | --- |
| **p** | Embedding dimension for non-seasonal time series. Number of non-seasonal lags used as inputs. | 1 to 20 by 1 | 6 |
| **P** | Number of seasonal lags used as inputs; size. | 1 to 10 by 1 | 8 |
| **decay** | Parameter for weight decay (“forgetting”). | 0.05 to 0.20 by 0.05 | 0.20 |
| **size** | Number of nodes in the hidden layer. | 1 to 3 by 1 | 1 |
